# Sociality as a Natural Mechanism of Public Goods Provision

**DOI:** 10.1371/journal.pone.0119685

**Published:** 2015-03-19

**Authors:** Elliot T. Berkman, Evgeniya Lukinova, Ivan Menshikov, Mikhail Myagkov

**Affiliations:** 1 Department of Psychology, University of Oregon, Eugene, Oregon, United States of America; 2 Skolkovo Institute of Science and Technology, Skolkovo, Russian Federation; 3 Department of Applied Mathematics and Management, Moscow Institute of Science and Technology, Dolgoprudny, Russia; 4 Department of Political Science, University of Oregon, Eugene, Oregon, United States of America; Hong Kong Baptist University, CHINA

## Abstract

In the recent literature, several hypotheses have been offered to explain patterns of human behavior in social environments. In particular, these patterns include ‘prosocial’ ones, such as fairness, cooperation, and collective good provision. Psychologists suggest that these prosocial behaviors are driven not by miscalculations, but by salience of social identity, in-group favoritism, emotion, or evolutionary adaptations. This paper imports psychology scholarship into an economic model and results in a sustainable solution to collective action problems without any external enforcement mechanisms. This natural mechanism of public goods provision is created, analyzed, and observed in a controlled laboratory environment using experimental techniques.

## Introduction

People’s economic decisions are often embedded in a social context. To what extent does that context influence their decisions, if at all? Social factors such as group memberships and affiliation motives have powerful effects on a range of behaviors. These factors carry substantial decision utility for people, but this “social utility” is rarely included in formal models of economic behavior. Here, we marry some of the rich models of social behavior taken from social psychology with decision modeling techniques from behavioral economics. Recent efforts to unite these two traditions have proven fruitful in delivering theoretical insights and model-based precisions for studying economic behavior in a realistic social context [1, 2]. Specifically, we use a classic “minimal group” paradigm from social psychology to induce a sense of social connectedness in our experimental subjects. It measures their degrees of utility conferred by their sociality which otherwise are found to not have economic utility.

Classical economic theory has been strongly challenged by findings where economic players often do not reason by pure utility-maximization techniques. The critical breakdown point of economic models is in explaining behaviors that are altruistic or at least non-selfish. The fact that human behavior is not driven solely by economic considerations is not intuitively surprising. Few people believe their motives to be entirely economic. Acts of “irrational” generosity to others at one’s own cost are rewarded through non-economic means such as a subjective sense of satisfaction and a conferral of social status from others. Notable patterns of human behavior that fit this mold—and that result in prosocial outcomes—include “economic irrationality” [3–5], sustainable cooperation [6–8], inequity aversion [9–11], and altruism [12, 13] in a social environment.

Humans have been described as “social animals” because our survival as a species as well as at the individual level depends on common group goals and collective action [14, 15]. From this perspective, the breakdown of cooperation predicted by economics [16–19] does not seem inevitable. Experimental studies explain the mechanism behind cooperation through reciprocity and conditional cooperation [20–23]. Darwinian evolution adds several mechanisms as explanations for cooperative behavior, such as kin and group selections, similarities among individuals, and indirect reciprocity through good reputation [23–26]. A “spatial reciprocity” mechanism can also promote cooperation under certain conditions [24, 27, 28]. However, spatial networks assume that actors interact with some individuals more often than others. The procedure and results presented in this manuscript do not rely on this assumption; each participant interacts with the others in a small population with relatively equal frequency, in which case the natural selection mechanism of defection is still expected to prevail [24, 28].

From an evolutionary perspective, only groups with a significant cooperation rate will be sustainable [29, 30]. In fact, humans evolved behavioral features that allowed them to detect cooperators and facilitate cooperation [31–33]. These prosocial behaviors are likely triggered by specific social environments characterized by an increased salience of one’s identification with the group (“social identity”). This saliency is defined as knowledge, value, and emotional significance of group membership [34]. Humans achieve a positive social identity through intergroup social comparisons and are able to distinguish between in-group and out-group, thus maintaining cooperation in the long run [35]. Social identity creates a sense of “in-group favoritism” that associates positive characteristics with the in-group members [36, 37]. It results in advantageous treatment of the in-group [38–40], greater cooperation with the in-group than with the out-group members [41, 42], and establishes fairness norms [43, 44]. In economic terms, social identity may be a key mechanism by which sociality comes to have positive decision utility.

Our overarching hypothesis is that sociality, even in a very minimal form, serves as a natural mechanism of sustainable cooperation. This has not yet been directly demonstrated empirically. Here, we report on a series of laboratory experiments that combine group-based manipulations of the social environment (i.e., sociality) with the possibility of sustained cooperation. In these experiments participants face each other in a social environment and play economic games, such as the Prisoner’s Dilemma and Ultimatum Game. The one-shot Prisoner’s Dilemma game provides participants with the opportunity for cooperation, and the Ultimatum Game can reveal egalitarian and altruistic strategies. If our hypothesis is correct, participants will behave more cooperatively, and do so in a more sustained way, in the socialization condition. The main results of the experiments confirm this expectation: the social environment creates not only short-term but also long-term cooperation and egalitarian preferences. The group-based social factors suggested by evolutionary and social psychologists are shown here to alter the expected patterns of economic behavior based solely on a motive to maximize one’s own immediate utility.

Next subsection of the paper outlines relevant theories and models of social utility and specifies the questions to be answered by the research. Section 2 details the experimental design and the methods that are needed to test the research questions. Section 3 contains the results. The final section summarizes and concludes the paper.

## Theory and Model for Behavior

For the purposes of this paper, sociality, or social utility, is defined as an additional component of the utility function. It reflects the value of contributing to group success derived from social identity. The idea that humans may care for more than their narrowly-defined material interest and that their decisions may be driven by sociality and other non-monetary considerations is not new to behavioral and experimental economists [45]. However, economists rarely consider utility functions that include social aspects [46]. If it is included at all, the social component is usually a function of the outcome and is viewed as strategic learning or just error [17–19] that dissipates with repetition.

On the other hand, social psychologists have studied social interactions for well over 50 years and suggest that decision-making changes under risk in a social environment. People can serve as means through which gains can be obtained or losses prevented [47] and, therefore, a separate function that accounts for social factors might come into play. Social factors might thus change not only observed risk attitudes for gains and losses, but, as suggested by Kahneman and Tversky [48], also make behavior prosocial. However, social psychologists rarely if ever formalize their findings of peculiar behavioral patterns or quantify the value of sociality.

Following on the breakthrough work by evolutionary psychologists [49], this paper emphasizes the importance of the substantive context of social decisions. A theory of sociality is developed, tested, and refined with the use of experimental methodology in the context of social setting. Sociality is modeled as an additional component of an individual’s utility function.

$$U_{i}=U^{0}{}_{i}(z)+U^{s}{}_{i}(z)$$ (1)

$U^{0}{}_{i}(z)$ is the individual utility function of an outcome; $U^{s}{}_{i}(z)$ is the individual social utility component; *z* is outcome; and *i*—individual. The latter is sociality, defined in this paper as the value of contributing to group success and informed by social psychology.

Group success and cooperation is viewed by social psychologists to be a function of the relative salience of social identity, or the part of one’s self-concept that derives from the knowledge, value, and emotional significance of group membership [34, 50]. Value is used to mean a personal priority, "I value family so I take time off of work to be with my children," and emotional significance is about the subjective feeling associated with the group, such as, "I enjoy being with my family". Those two things are not necessarily the same—one might feel a sense of moral obligation to one's family (a value) but derive no pleasure from it (value without emotion). On the other hand, one might always enjoy being with family but not feel any priority towards family (emotion without value). Alike, the value of contributing to group success in the model presented here depends on two factors that can increase individually or together with socialization. Sociality captures the value of encountering a cooperator, the positive affect associated with encountering another cooperator, the satisfaction and sense of common identity that comes from working towards group success, or an emotional significance of group membership in psychological terms. The value of contributing to group success also depends on tolerance of a certain amount of defection from in-group members, presumably reflecting a sense of moral obligation to their group. In other words, the personal priority to achieve group success or to bolster the common sense of group identity allows a person some tolerance for free-riding behavior.

The research questions of this paper are whether sociality helps (a) promote more cooperative and fair choices, (b) maintain good equilibria, (c) is larger for cooperators than for defectors (to allow for the cooperators to recover from being defected on), and (d) allows punishing individuals without punishing the whole group. We make three core assumptions in this model. First, sociality exists only if a participant cooperates. Second, fair offers, e.g., an offer of 5 out of 10, become a moral obligation for participants and if an unfair offer happens, e.g., an offer of less than 5 out of 10, it cannot be tolerated and might be rejected. This is a perfect example of punishing the individual but not the group. Finally, sociality depends on components that encourage cooperation and tolerate defection, and increasing the degree of socialization will make the utility to cooperate large enough to catalyze a new equilibrium.

$$U_{i}=U^{0}{}_{i}(z)+V^{s}{}_{i}$$ (2)

$V^{s}{}_{i}$ is sociality or value of contributing to group success created through socialization; *z* is an outcome; and *i*—individual.

*Assumption 1*: The additional component of the utility function is activated only if a participant cooperates or is fair, and thereby works toward group success.

$$\{\begin{array}{c} V^{s}{}_{i}=0,\begin{array}{ccc} & if & D\begin{array}{ccc} & or & NF \end{array} \end{array}\\ V^{s}{}_{i}>0,\begin{array}{ccc} & if & C\begin{array}{ccc} & or & F \end{array} \end{array} \end{array}$$ (3)

where *D* is defection, *C*—cooperation, *F*—a fair offer, and *NF—*an unfair offer.

*Assumption 2*: The value of contributing to group success depends on two components: the value of encountering cooperator (*a*_*i*_) and an ability to tolerate defection (*b*_*i*_)

$$EV^{s}{}_{i}=a_{i}p+b_{i}(1-p)$$ (4)

$EV^{s}{}_{i}$ is the expected value of contributing to group success; *p* is the probability of cooperation or a fair offer. As a first approximation, we assume this function to be linear for clarity and simplicity.

$$\{\begin{array}{c} V^{s}{}_{i}=a_{i},\begin{array}{ccc} & if & opponentchooses\begin{array}{ccc} & C & \end{array}\begin{array}{ccc} & or & F \end{array} \end{array}\\ V^{s}{}_{i}=b_{i},\begin{array}{ccc} & if & opponentchooses\begin{array}{ccc} & D & \end{array} \end{array}\begin{array}{ccc} & or & NF \end{array} \end{array}$$ (5)

*Assumption 3*: Both components are strictly greater than zero: *a*_*i*_>0, *b*_*i>*_0_,_ *a*_*i*_ *>b*_*i*_

*Proposition 1*: Both components (*a*_*i*_) and (*b*_*i*_) are positively correlated with the value of contributing to group success.

*Proof*: Our model states that sociality, or the value of contributing to group success, is a linear combination of (*a*_*i*_) and (*b*_*i*_). As 0 < *p* < 1 in Equation 4, with increase in each of the components the value of contributing to group success will increase as well, i.e. $V_{i}^{S}>V_{i}^{0}$.

*Proposition 2*: Cooperation declines if $\sum_{i}V_{i}^{s}<Threshold$.

*Proof*: Let us define the *Threshold* as the critical value for an average individual *i* to cooperate. If $V^{s}{}_{i}$ is smaller than the *Threshold*, then the utility to cooperate is smaller than the utility to defect and the utility of making a fair offer is smaller than making an unfair offer for at least half of the participants. We assume that out of all individuals, some always defect, some always cooperate, and others use certain rules to decide on their strategy. Thus, in sum, there are not enough individuals to overcome the Nash Equilibrium of the PD and UG.

*Proposition 3*: Socialization maintains long-term cooperation if $\sum_{i}V_{i}^{s}\geq Threshold$.

*Proof*: Before socialization $V^{0}{}_{i}$, the value of contributing to the group success, is not high enough and cooperation declines. After socialization, this component of the utility function makes cooperation sustainable if it is larger than the *Threshold* for enough individuals *i*. This means that at least some of the individuals cooperate and more so than before socialization. The model states that sociality *V* ^*s*^_*i*_ becomes larger together with its components *a*_*i*_ and *b*_*i*_. If $V^{s}{}_{i}$ is large enough, so that cooperation is the dominant strategy in the first round for some individuals, then they will not switch from their dominant strategy at a later round.

It is assumed that total utility for a participant depends on the utility of an outcome and on sociality (Equation 2). However, the nature of the choices in the Prisoner’s Dilemma (PD) and in the Ultimatum Game (UG) is different. Both games used in this study are one-shot games, not iterated. Although multiple rounds of these games are played, each round (one-shot) is played with a computer or a random anonymous human opponent. Whereas PD is a simultaneous one-shot game that does not allow playing Tit-for-Tat and not punish the group, UG is a sequential one-shot game that allows using Tit-for-Tat, i.e., to decline the unfair offer and punish the individual without punishing the group. Thus, the propositions concerning PD and UG vary.

For cooperation in PD to be sustainable the expected utility to cooperate should exceed the expected utility to defect:

$$EU_{i}(C)>EU_{i}(D)$$ (6)

The key here is that the new component of the utility function, sociality, becomes large enough to overcome the PD Nash equilibrium in defection. The expected utility to cooperate now consists of the individual expected utility and $V^{s}{}_{i}$– the value of contributing to group success:

$$\begin{array}{l} EU_{i}(C)=p_{c}u(C,C)+(1-p_{c})u(C,D)+p_{c}a_{i}+(1-p_{c})b_{i}>\\ >EU_{i}(D)=p_{c}u(D,C)+(1-p_{c})u(D,D) \end{array}$$ (7)

where *p*_*c*_ is probability of cooperation in a group, *u(C*,*C)*—is the outcome of individual that cooperates and receives cooperation, *u(C*,*D)*—is the outcome of individual that cooperates and receives defection from his opponent, *u(D*,*C)*—is the outcome of individual that defects and receives cooperation, and *u(D*,*D)*—is the outcome of individual that defects and receives defection from his opponent.

*Hypothesis 1*: For the UG only one component is strictly greater than zero.

*a*_*i*_ *>b*_*i*,_ *a*_*i*_>0, *b*_*i*_ = 0

$$EV^{s}{}_{i}=a_{i}p_{a}$$ (8)

*p*_*a*_ is the probability of accepting an offer in a group.

For fairness in the UG to be sustainable, the expected utility to offer a fair amount (an offer of 5 out of 10 is considered to be fair) should exceed the expected utility to offer an unfair amount:

$$EU_{i}(F)>EU_{i}(NF)$$ (9)

The key here is that the new component of utility function, sociality, becomes large enough to overcome the UG Nash equilibrium of offering anything bigger than zero. The expected utility to cooperate now consists of the value of encountering a cooperator (*a*_*i*_), or a person, who offers fair amounts, whereas defection, or making unfair offers, is no longer tolerated.

$$\begin{array}{l} EU_{i}(F)=p_{a}\times (10-offer_{F}^{s})+(1-p_{a})\times 0+p_{a}a_{i}>\\ >EU_{i}(NF)=p_{a}\times (10-offer_{NF}^{s})+(1-p_{a})\times 0 \end{array}$$ (10)

*Hypothesis 2*: Sociality, the value of contributing to group success, increases with socialization, i.e. $V_{i}^{S}>V_{i}^{0},$ where $V_{i}^{0}\geq 0$ is the sociality before socialization.

*Hypothesis 3*: Socialization increases cooperation and egalitarianism. If 0 ≤ *p*_*c*_^*0*^ ≤ 1 is the cooperation rate before socialization and 0 ≤ *p*_*c*_^*s*^ ≤ 1 is the cooperation rate after socialization, then *p*_*c*_^*s*^> *p*_*c*_^*0*^. With an increase in the rate of cooperation, the value of encountering a cooperator (*a*_*i*_) also increases.

If 1 ≤ *offer* ^*0*^ ≤ 5 is the average offer in UG before socialization and 1 ≤ *offer* ^*s*^ ≤ 5 is the average offer after socialization, then *offer* ^*s*^ > *offer* ^*0*^.

*Hypothesis 4*: Socialization increases the tolerability of defection. If *b* ^*0*^ ≥ 0 is the tolerability to defection before socialization and *b* ^*s*^ ≥ 0 is the tolerability to defection after socialization, then *b* ^*s*^ > *b* ^*0*^. Our model states that sociality increases with socialization, i.e. $V_{i}^{S}>V_{i}^{0}.$ This means that, at least for some individuals, *b* increases with socialization.

*Hypothesis 5*: Socialization increases in-group favoritism. In-group favoritism is created through the salience of social identity, which can be measured directly using implicit and explicit association tasks and indirectly through the revealed value of contributing to group success, $V_{i}^{S}.$ This parameter increases with socialization according to the assumptions of the model.

Nevertheless, for sociality to appear and to sustain collective action, the individual’s membership in the group needs to be made salient. We achieved this here by creating a Socialization Phase that is detailed in the next section. Implicit and explicit tests of association of oneself with a group (from the social psychological literature) are used as manipulation checks to ensure that sociality is activated to a greater degree in the experimental group versus the control group.

## Materials and Methods

The study procedures involving human participants were approved by Skolkovo Institute of Science and Technology Human Subjects Committee. Written informed consents were obtained from participants. Experimental data are readily available on Figshare: http://dx.doi.org/10.6084/m9.figshare.1299951.

Subjects (N = 96, 32 females, Age M = 19) for the experiment were recruited from the students at the Moscow Institute of Physics and Technology (MIPT). The MIPT Experimental Economics laboratory was used to carry out all experiments. Each experiment consisted of 12 students, pre-selected before the experiment to be unfamiliar with one another. After the end of each treatment, participants provided feedback about the experiments received payments and left the experimental facility.

Each experiment was divided into 5 phases:

1. Computer Opponent phase, where participants played the Prisoner’s Dilemma (PD) (Table 1) and Ultimatum Game (UG) with a computer using a Nash equilibrium strategy each round: 0.85 probability for PD; uniform distribution [1, 5] as offer for UG (out of 10).
2. People Game phase, where participants played the PD (Table 1) and UG with a random human partner. Participants were randomly paired with an anonymous partner each round of the game and alternated roles on subsequent trials between column chooser and row chooser for the PD. Participants made offers (how many units to give to the partner out of 10) and then viewed the decision on the offer (accept, decline) for UG.
3. Socialization phase, where participants were divided into two groups of 6, got to know each other in pairs at first then in the larger group, and then completed the Group Game task (below). The socialization phase consisted of three main tasks. First, each participant got to know another participant from his/her group, and then each small group of two narrated to the group of 6 about his/her partner’s characteristics and life facts. Finally, the group of 6 completed the task of identifying five characteristics that everyone in their group shares, and then selected one of those characteristics as their group’s name. The group then provided to the experimenter a list with the characteristics written down and the group’s name circled.
4. Group Game phase, where participants played the PD and UG with a random human partner from their group of 6. Their partner changed each round of the game. The participants switched roles on alternating trials: column chooser and row chooser for PD; offerer and decision-viewer on the offer (accept, decline) for UG. The games were the same as in People Game phase. The Group Game phase lasted 15 rounds for most of the experiments. However, we did a couple of experiments with 20 rounds and tracked the feedback of the participants. Unlike the feedback for the experiments with 15 rounds, these participants noted being bored towards the end of the experiment and made their responses automatic. That said we believe that our decision to keep 15 rounds was valid, because the decisions of participants were thought through and, thus, could be trusted.
5. Finally, a Manipulation Check was acquired using the Implicit Association Test [51] and an explicit questionnaire that measured the extent of participants’ group identity on a 7-point Likert scale. The IAT measures the strength of association between concepts by observing reaction times in categorization tasks. First, participants classified photographs of participants into group membership (in-group / out-group) using two buttons indicated in the task. Then, participants classified pictures (smiley and non-smiley faces) into valence categories (pleasant / unpleasant) using the same two buttons. Lastly, in the critical phase, photographs and pictures were combined into two new response time classification tasks with in-group + positive / out-group + negative categories or out-group + positive / in-group + negative as the groupings, presented in counterbalanced order. The difference in average reaction time between two combined tasks in the critical phase yields the IAT measure.

**Table 1:** Prisoner’s Dilemma Payoffs.

	Cooperate	Defect
Cooperate	5,5	0,10
Defect	10,0	1,1

Numerical values for Prisoner’s Dilemma are chosen to be comparable with Ultimatum Game, with the division of the pie of 10.

The socialization treatment is the standard experiment as described above, and the control treatment was exactly the same except without the socialization phase. Other modifications of the experiment include an Established Groups treatment that enrolled participants from two different communities and an Auction treatment (S1 Text1). It is assumed that in the Established group condition there will be even bigger increase in cooperation and egalitarianism as participants are socialized in their communities for a longer time. However, another result is also possible. Once you know the group well enough, you know who are likely to engage in good behaviors and who are not, thus, one might as well see a decline in the cooperative and egalitarian strategies. This treatment was attempted once, but due to discrepancy in groups appearance to the experiment was excluded from analysis.

The Computer Opponent phase included 10 rounds of PD (Table 1) and 10 rounds of UG (decision on division of 10 units). In our preliminary study we used a different payoff matrix, i.e. 1<2<4<6, instead of 0<1<5<10, and reached the same results as in this manuscript. Both the People Game and Group Game phases included 15 rounds of PD (Table 1) and 15 rounds of UG (decision on division of 10 units). A round was a one-shot game (either PD or UG with a computer or random anonymous human opponent). The PD was played simultaneously, so that the partners in the game see the decision screen and make a decision to Cooperate or Defect. Only when both partners made their choices did they both see the outcome screens indicating how many points they earned for the current round. The UG was played sequentially: first, the player who offers a division typed in and submitted an offer (how many units to give to the partner out of 10), and then the partner observed the offer and decided whether to accept (division is done according to the offer) or reject (both partners get zero points) the offer. Only then did both partners see the outcome screens. The order of the experimental phases followed the sequence described in the Experimental design section and remained unchanged for all experiments. Participants do not know each other at the beginning of the study and played the games as strangers at first, and then got to know each other (socialization) and played within their groups. The total duration of the experiment did not exceed two hours.

The research questions of this study are (a) whether the Socialization phase creates sociality and in turn (b) whether that socialization serves as a natural mechanism of collective goods provision or a good equilibrium, in which long-lasting cooperation and fairness are established.

## Results

### Result 1: Socialization increases cooperation and fairness and sustains this equilibrium

Cooperation rates in the Group Game phase on average differed significantly from those in the People Game phase as shown in Table 2. In fact, cooperation and the value of encountering a cooperator (*a*_*i*_) not only increased in Group Game phase but also maintained throughout the course of trials to a greater degree than in the People Game phase. Table 3 depicts the same information for the socialization and control treatments of all experiments.

**Table 2:** Prisoner’s Dilemma (Cooperation Rate).

	First 5 periods	5–15 periods	All
People	32%	24%	27%
Groups	48%	47%	47%

Cooperation rates in Prisoner’s Dilemma are significantly higher for Group Game phase than for People Game phase (n = 90, P-value = 0.0075, t-test). There is no significant difference between rates for Group Game phase during the first 5 periods if compared to 5–15 periods.

**Table 3:** Prisoner’s Dilemma (Cooperation Rate).

		First 5 periods	5–15 periods	All
03102013	People	23%	14%	17%
	Groups	30%	27.5%	28%
05102013	People	25%	10%	15%
	Groups	32%	21%	24%
12102013	People	23%	14%	17%
	Groups	33%	33%	33%
19102013_1	People	42%	22%	29%
	Groups	83%	82.5%	83%
19102013_2	People	38%	36%	37%
	Groups	63%	61%	62%
26102013	People	43%	35%	38%
	Groups	52%	47%	48%
16112013 (Control)	People	28%	17.5%	21%
	Groups	58%	53%	55%
23112013 (Control)	People	25%	17%	19%
	Groups	47%	21%	29%

For all experiments cooperation rates in Group Game phase were significantly higher (n = 90, P-value = 0.000, t-test).

Fairness increased with socialization (Table 4). There were significantly more fair offers (defined as at least 5 out of 10) in the Group Game phase than in the People Game phase (n = 90; P-value = 0.0098, t-test).

**Table 4:** Ultimatum Game (Offers).

	5	4	3	2	1
People	24%	45%	21%	5%	5%
Groups	44%	38%	11%	4%	3%

There was a significantly higher number of fair offers (an offer of 5 out of 10) in Group Game phase than in People Game phase (n = 90, P-value = 0.0098, t-test)

These results support the third hypothesis that socialization increases cooperation and fairness. In contrast, in two control experiments the increase in cooperation from the People Game phase to the Group Game phase was not significant. We note that in both the socialization and control treatments there were idiosyncratic group effects due to the difference in the quality of the socialization, and that these group differences are accounted for in our model in terms of a group constant as a part of the social utility equation.

### Result 2: Socialization increases the value of contributing to group success

In general, socialization significantly increased cooperation rates and their stability in the Group Game phase compared with the People Game phase (Table 2 and Table 3). This suggests that for most of the experiments the value of contributing to group success exceeded a strict rational according to the classical economic theory.

**Fig 1:**
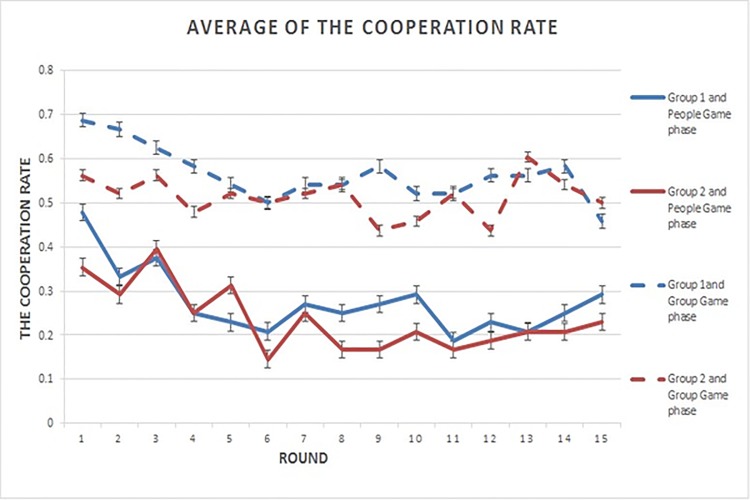
Average of the Cooperation Rate. The cooperation rate (y-axis) as a function of round or time (x-axis). Solid blue lines indicate average cooperation rate over time for Group 1 and People Game phase (average is taken for 6 experiments (socialization treatment)), solid red lines—for Group 2 and People Game phase, dotted blue lines—for Group 1 and Group Game phase, and dotted red lines for Group 2 and Group Game phase. Error bars represent standard error (SE).

To more closely examine and account for group specific effects, we further analyzed 6 experiments (socialization treatment) containing a total of 12 groups. Of these, 9 out of 12 groups evinced only a slight to no decline in cooperation. The average data for Group 1 and Group 2 is shown in Fig. 1. In this figure the dotted lines for Group Game phase lie above smooth lines for People Game phase of the same color, suggesting greater cooperation in the Group Game phase. Also Table 5 displays the negative correlations between the decline of cooperation and group identification level, measured by the explicit (i.e., self-reported) test. This supports the second hypothesis that cooperation would be maintained on the group level as a function of socialization.

**Table 5:** Dependence of Decline in Cooperation on Group Identification Level.

Group #	1	2	3	4	5	6	7	8	9	10	11	12
Group Identification	4.2	4.5	2.7	3.3	4.2	3.7	5.7	3	3.7	5	4.2	3.5
Decline in Cooperation	0.1	-0.05	0.15	0.07	0	0	-0.07	0.13	0.2	-0.15	0.083	0.017

Slight negative correlation (-0.43) is present between decline in cooperation and group identification level, measured by the explicit (i.e., self-reported) test.

Next, we analyzed individual participant data (72 from 6 experiments of socialization treatment) by dividing all socialized participants into four categories as displayed in Table 6. Type 1 are participants who never cooperated; Type 2 are participants who cooperated more in People Game phase than in Group Game phase; Type 3 are participants who cooperated less in People Game phase, than in Group Game phase; and Type 4 are participants who only cooperated.

**Table 6:** Types of Participants in Percentages.

	Type 1	Type 2	Type 3	Type 4
Number of Participants	11	14	46	1
Percentage	15%	19%	64%	1%

Type 1 are participants who never cooperated; Type 2 are participants who cooperated more in People Game phase than in Group Game phase; Type 3 are participants who cooperated less in People Game phase, than in Group Game phase; and Type 4 are participants who only cooperated.

To test the second hypothesis on the individual level participants of all types are considered. The question is: do more participants maintain their cooperation through the last period in the Group Game phase than in the People Game phase? For this purpose, we defined an index of change in the level of cooperation as the difference between the average cooperation rate for all periods and for the last five rounds. This index equals zero for participants of Type 1 and Type 4, because they either cooperate in all rounds or do not cooperate at all, thus, no change from first rounds to the last ones. This index is negative when cooperation increases in the last five rounds from the average level.

**Fig 2:**
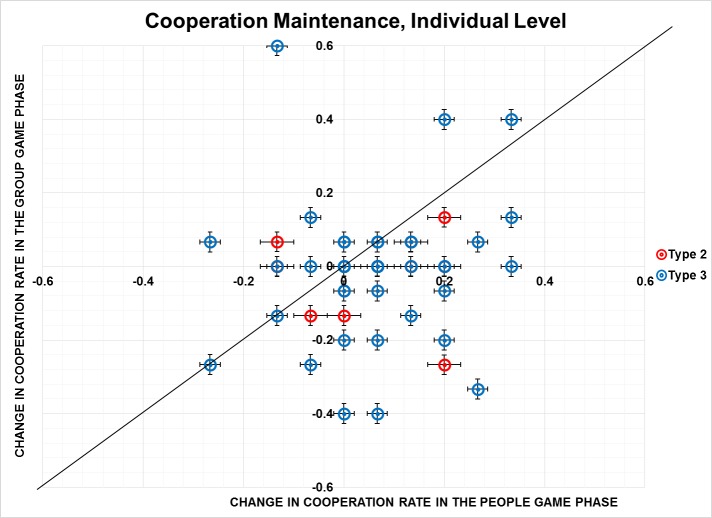
Cooperation Maintenance, Individual Level. The change in cooperation rate (from later to the earlier round of the game) for the Group Game phase (y-axis) as a function of change in cooperation in the People Game phase (x-axis) for Type 2 and Type 3 individuals. Type 2 are participants who cooperated more in People Game phase than in Group Game phase; Type 3 are participants who cooperated less in People Game phase, than in Group Game phase. Each square is a participant of Type 2, each triangle—participant of Type 3. Error bars represent standard error (SE).

Fig. 2 plots change in the level of cooperation in the People phase (x-axis) against change in cooperation in the Group Game phase (y-axis) for Type 2 and Type 3 individuals. Most of the dots lie below y = x, and, in particular, in the fourth quadrant, which means that cooperation is stable or increases slightly during the Group Game phase compared to the People phase towards the end of experimental periods among participants of Type 2 and Type 3. A couple of outliers (i.e., the dots on the top) decreased in their cooperation faster in the Group Game phase. Nonetheless, on average, the second hypothesis cannot be rejected on all levels of the analysis. Socialization not only creates more cooperation, but also maintains it better.

### Result 3: Socialization increases the tolerance for defection

The fourth hypothesis is that socialization increases the tolerability of defection. Our data reveal directly opposite results on this hypothesis for the Ultimatum Game and the Prisoner’s Dilemma. In the Ultimatum Game this hypothesis is rejected, because agreement to unfair offers declines slightly with Socialization (Table 7). This, in turn, supports the first hypothesis that sociality keeps the ability to tolerate defection equal to zero.

**Table 7:** Ultimatum Game (Acceptance).

	5	4	3	2	1
People	100%	85%	44%	20%	0%
Groups	100%	77%	32%	0%	0%

There was not a significantly higher number of acceptances in Ultimatum Game in Group Game phase than in People Game phase. (n = 90, P-value = 0.779, t-test)

In the Ultimatum Game participants could agree or not agree to the offer their opponent made and therefore had an opportunity to punish the opponent. In contrast, the Prisoner’s Dilemma is a one shot game and thus does not allow punishment. To measure responses to defection in the PD, we defined a Bearable defection (BD) index as the maximum defects one receives in the PD from a partner before switching from cooperation to defection. This index reflects the *b*_*i*_ component of sociality, the ability to tolerate defection.

Across all experiments (6 experiments with socialization treatment) and summing up BD indexes of participants of Type 2 and Type 3 (because the other types did not switch their strategy) there is a visible increase in tolerance for defection with socialization:

BD (People) = 87 < BD(Group) = 119 (n = 12, P-value = 0.0475, chi-squared).

In parallel on the group level, 8 out of 12 groups experienced an increase in tolerability, 1 group’s tolerability didn’t change, 3 groups showed a decline in tolerability. Table 8 depicts Bearable defection indexes for the Group Game and the People Game phases for each of the 12 groups. The null hypothesis, independence of group number and tolerance with and without socialization, cannot be rejected, because the chi-squared test value 12.358 is less than critical value 19.675.

**Table 8:** Bearable Defection by Group.

Group #	BD(People)	BD (Group)	Total
1	7	15	22
2	4	9	13
3	7	7	14
4	6	10	16
5	6	13	19
6	7	3	10
7	5	1	6
8	10	18	28
9	5	8	13
10	7	10	17
11	19	18	37
12	4	7	11
Total	87	119	206

To measure responses to defection in the PD, we defined a bearable defection (BD) index as the maximum defects one receives in the PD from a partner before switching from cooperation to defection. This index reflects the bi component of sociality, the ability to tolerate defection. Across all experiments (6 experiments with socialization treatment) and summing up BD indexes of participants of Type 2 and Type 3 (because the other types did not switch their strategy) there is a visible increase in tolerance for defection with socialization:

BD (People) = 87 < BD(Group) = 119 (n = 12, P-value = 0.0475, chi-squared).

For the individual level analysis, all participants of Type 1, some participants of Type 2 and Type 3 had to be excluded, because they never had a chance to bear defection. The rest form the sample for hypothesis testing. The number of participants of each type is shown in Table 9. The proportions of homogeneity between all participants and the approved sample is tested using chi-square test. Homogeneity is rejected with a test value 12.993 bigger than critical value 9.488. A comparison of Bearable Defection indices (Table 10) shows both the percentages of all participants and of the approved samples for which the inequality holds.

**Table 9:** Number of participants.

Number of participants	Overall (A)	Approved Sample (S)
Type 2	14	6
Type 3	46	33
Type 3 BD(G)>BD(P)	25	20
Type 3 BD(G) = BD(P)	10	9
Type 3 BD(G)<BD(P)	9	4

The proportions of homogeneity between all participants and approved sample is tested using chi-square test. Homogeneity is rejected with test value 12.993 bigger than critical value 9.488.

**Table 10:** Individual BD Differences.

Type	Type 2 (A)	Type 3 (A)	Type 2 (S)	Type 3 (S)
BD (Group)>BD (People)	0%	57%	0%	61%
BD (Group) = BD (People)	0%	23%	0%	27%
BD (Group)<BD (People)	100%	20%	100%	12%

Bearable Defection indexes comparison shows both the percentages of all participants and of the approved sample for which the inequality holds. Participants of Type 2 are less cooperative with socialization and they can bear less defection in Group Game phase as well. Socialization for participants of Type 3 not only increases cooperation rate, but also allows them to bear more defects from their opponents. (A) stands for all participants, (S) stands for Approved Sample.

The fourth hypothesis can be rejected for participants of Type 2. These participants are less cooperative with socialization and they also bear less defection in Group Game phase. For participants of Type 3 this hypothesis cannot be rejected. Socialization for them not only increases cooperation rate, but also allows them to bear more defections from their opponents.

### Result 4: Socialization increases explicit group identification

Implicit and explicit self-with-group associations are used as an index of in-group favoritism. The effect of Implicit Association Test is measured with a D-score that has a possible range of-2 to +2. Break points for ‘slight’ (.15), ‘moderate’ (.35) and ‘strong’ (.65) association effect are selected conservatively according to conventions for IAT effect size [51].

**Fig 3:**
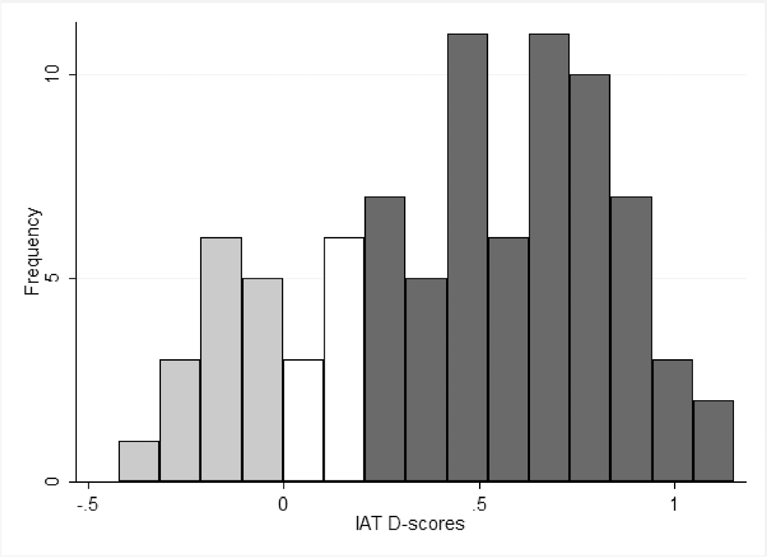
IAT D-scores. The histogram of 84 IAT D-scores (x-axis) for the group association task completed in the end of each experiment. The dark bars indicate faster sorting of out-group with Unpleasant and in-group with Pleasant, gray bars indicate faster sorting of in-group with Unpleasant and out-group with Pleasant. The bar height (y-axis) indicates the number of people who scored within that range.

The distribution in Fig. 3 summarizes 84 IAT D-scores (8 experiments: socialization treatment and control treatment) (mean = 0.456) for the group association task completed at the end of each experiment. There is no observed difference in the D-score mean from the experiment with Socialization phase to the experiment without Socialization phase. Therefore, the observed D-scores do not support our argument that the socialization was sufficient to induce a sense of group identity. It is noteworthy that, though implicit (e.g., the IAT) and explicit (e.g., self-reported group identity) tests are generally related, they might not measure the same thing and, thus, tend to have a low correlation with each other [52, 53].

**Fig 4:**
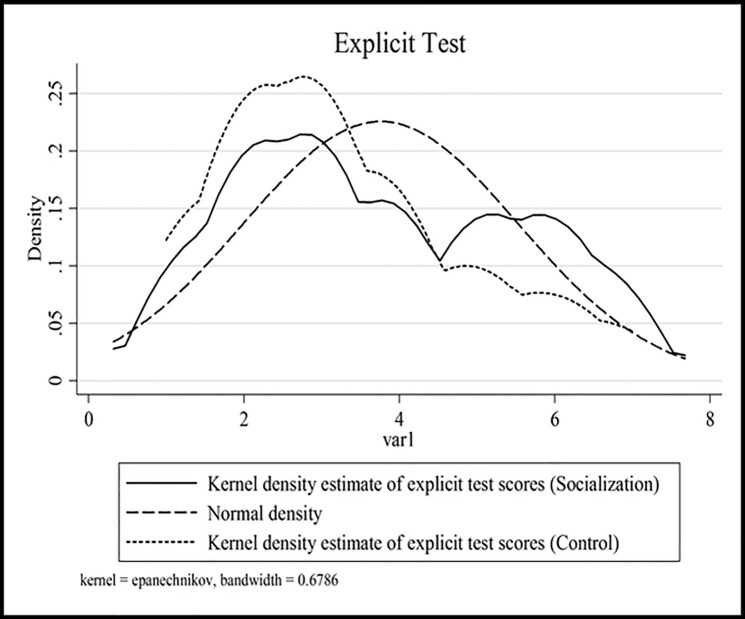
Explicit Test. Kernel density estimates of explicit test scores. Solid line—for experiments with Socialization, dotted—for Control, dashed—for Normal distribution density.

A comparison of explicit test scores between the Socialization and Control treatments suggests a slight increase in the self-report measure of identification with the group and a significance difference in adjusted scores (Table 11). Adjusted scores are formed by excluding scores that do not correspond to the feedback that participants gave at the end of experiment. Fig. 4 shows a difference among the kernel density estimates of the explicit test scores.

**Table 11:** Explicit Test Average Scores.

	Socialization Treatment	Control Treatment	All
All	3.76	3.25	3.63
Adjusted by Discrepancy with Feedback	3.75	3	3.56

A comparison of explicit test scores between Socialization and Control treatments suggests a slight increase in the score of identification with the group and significance difference for adjusted scores (N = 96, P-value = 0.0532, t-test), which are formed by excluding scores that do not correspond to the feedback provided at the end of experiment.

Although the implicit test rejects the fifth hypothesis, the explicit test and the changes of behavior with socialization support it. First, there is a significant increase in cooperation and egalitarianism for the Group Game phase, where participants play with the people with whom they socialized. In the Socialization treatment there is clear in-group favoritism as demonstrated by the increased and sustained cooperation patterns. In fact, cooperation rates are correlated with explicit test scores, even though the patterns of egalitarianism are not correlated with in-group salience. This may well reflect the differences between the two games under consideration: a one-shot Prisoner’s dilemma game, *without* a possibility to punish an individual opponent, versus an Ultimatum game *with* the possibility of punishing an opponent while not hurting the group as a whole.

## Discussion

This manuscript has developed a framework and model for studying individual decisions made in a social context, thereby creating a bridge between economics and social psychology. The results of the experiments were broadly consistent with our predictions: sociality increases and maintains cooperation and fairness, thereby providing a specific mechanism by which social groups can overcome the Nash equilibrium and sustain collective action.

First, socialization not only created cooperative equilibrium, but also maintained it, resulting in a sustainable solution of collective action without any external enforcement mechanisms. Second, the social group manipulations created equality and society, where one can punish a free-rider without punishing the group as a whole. Finally, we verified that sociality was made salient using a socialization induction.

Future work could capitalize on recent advances in neuroimaging to test whether patterns of brain activation during economics games with and without socialization replicate the behavioral results presented here and, importantly, establish the underlying neural mechanisms of the observed behavioral effects. Similar to this research, neuroscience scholars have suggested that some important behaviors may be aimed at maximizing social, not personal material outcomes [54]. Neuroimaging is seen as a critical tool for understanding the nature of the various aspects of human behavior, and recent trends in the field have placed particular emphasis on social behavior [55]. Use of this methodology has the potential to advance empirical evidence relevant to existing theoretical accounts of how people make decisions by informing and constraining these models based on the underlying neuroscience.

## Supporting Information

S1 Text Auction Treatment. The description of additional treatment used in the study. Its main purpose was to measure sociality.
(DOC)
