# Supplementary material for: Sociality as a Natural Mechanism of Public Goods Provision
Source: PLoS One. 2015 Mar 19;10(3):e0119685. doi: 10.1371/journal.pone.0119685 (PMC4366235; doi:10.1371/journal.pone.0119685)
Supplement: S1 Text — The description of additional treatment used in the study. Its main purpose was to measure sociality. (DOC) [file pone.0119685.s001.doc]

**Text S1**

**Auction Treatment**

In order to measure sociality in the modified treatment (Auction treatment, In-group/ Out-group) an auction is included in the Group Game phase. Auction is held once in 5 rounds and decides who is included in which of the 3 groups of 4 for all 5 rounds. Participants name a percent of their outcome they are willing to pay to play with their own group. The pay is taken only in the 1st out of 5 rounds, calculated from the outcome of this round. To win the auction and play in your group you need to be among four people in your group with the biggest percentages. There are two groups of 4, where each consists of players from one group and one mixed group of 4 with two players from one group and two players from another group that bid the least in the auction.

Sociality is modeled as an additional component of individual’s utility function, a function of individual and group specific variables:

$$U_{it}=U_{it}(z)+f(s_{it},s_{gt}),$$

where *z* is outcome; *s* – sociality or social value: $s_{it}$ - social value of an individual at given period, $s_{gt}$ - social value of a group at given period; *i* – individual; *t* – time; *g* – group.

Sociality helps promote more cooperative and fair choices, maintain good equilibria, is larger for cooperators than for defectors (to allow for the cooperators to recover from being defected on), and allows punishing individuals without punishing the whole group. The individual specific variable can be derived from each participant’s behavior and is positively correlated with the rate of cooperation. A group specific variable also is needed to model the differences between the groups in quality of socialization. It is formed by uniting an explicit test of group association results with feedback provided after the experiment.

Social value functional form is determined with the use of regression techniques. In-group/ Out-group treatment provides the sociality value variable (*Bid_t_*) as a percentage paid for in-group game which is regressed on individual, group constants, and their interaction.

$$Bid_{t}=\alpha s_{it}+\beta s_{gt}+\gamma(s_{it}\times s_{gt})+\varepsilon_{it}$$

*Auction Treatment Varieties*

1. Auction phase is included right after a short Group Game phase (5 rounds). Participants need to tell how many points they want to have not to play the game. Then, the ratio (ratio=bid/expected value from the last games) is calculated. Four participants (2 participants out of each group in Group Game phase) with the least ratio get the points and not play the game in this round. Pilot experiments portray that this version drives away cooperation and allows greedy participants to get the biggest profit.
2. Auction phase is included both before each round of People Game phase and Group Game phase. Participants need to tell how many points they want to have not to play the game. Four participants (4 participants in People Game phase and 2 participants out of each group in Group Game phase) with the least bids get the points and not play the game in this round. Pilot experiments reveal that bids do not reflect desire to play in group, in fact, they reflect exactly the opposite, driving down the value of the group.
